# Supplementary material for: Implementation of field detection devices for antimalarial quality screening in Lao PDR—A cost-effectiveness analysis
Source: PLoS Negl Trop Dis. 2021 Sep 30;15(9):e0009539. doi: 10.1371/journal.pntd.0009539 (PMC8483304; doi:10.1371/journal.pntd.0009539)
Supplement: S1 Text — Text A. List of information acquired from manufacturers for device cost estimation. Table A. Median total time taken per sample in sample set testing. Fig A. Results of sensitivity analyses from the cost-effectiveness analysis. Table B. Results from sensitivity analyses with lower detection rate with visual inspection (A) 12.5% and 25% reduction for substandard and falsified medicines, respectively and (B) 5% and 10% reduction under both high and lower prevalence scenarios. Table C. Results from a sensitivity analysis when using one device per province instead of one per district per device in high prevalence scenario with a 1-sample strategy compared with visual inspection. (DOCX) [file pntd.0009539.s001.docx]

**Supporting information**

**S1 Text Additional information for methods and results**

**Text A. List of information acquired from manufacturers for device cost estimation.**

**Table A. Median total time taken per sample in sample set testing.**

**Fig A. Results of sensitivity analyses from the cost-effectiveness analysis.**

**Table B. Results from sensitivity analyses with lower detection rate with visual inspection (A) 12.5% and 25% reduction for substandard and falsified medicines, respectively and (B) 5% and 10% reduction under both high and lower prevalence scenarios.**

**Table C. Results from a** **sensitivity analysis when using one device per province instead of one per district per device in high prevalence scenario with a 1-sample strategy compared with visual inspection.**

**Article 4 in Medicine Quality Screening Devices Collection ‘A multi-phase evaluation of portable screening devices to assess medicines quality for national Medicines Regulatory Authorities’**

**Title: ‘Implementation of Field Detection Devices in Medicine Quality Screening in Lao PDR – A Cost-Effectiveness Analysis’**

**Authors and Affiliation**: Nantasit Luangasanatip^1^, Panarasri Khonputsa^1^, Celine Caillet^2,3,4^, Serena Vickers^2,3,4^, Stephen Zambrzycki^5^, Facundo M Fernández^5^, Paul N Newton^2,3,4^, Yoel Lubell^1,3^

^1^ Mahidol-Oxford Tropical Medicine Research Unit, Faculty of Tropical Medicine, Mahidol

University, Bangkok, Thailand.

^2^ Lao-Oxford Mahosot Hospital Wellcome Trust Research Unit, Microbiology laboratory, Mahosot Hospital, Vientiane, Lao PDR

^3^ Centre for Tropical Medicine and Global Health, Nuffield Department of Clinical Medicine, University of Oxford, United Kingdom.

^4^ Infectious Diseases Data Observatory (IDDO)/Worldwide Antimalarial Resistance Network (WWARN), University of Oxford, United Kingdom.

^5^ School of Chemistry and Biochemistry, Georgia Institute of Technology, Atlanta, Georgia, USA.

**Text A. List of information acquired from manufacturers for device cost estimation.**

| **Items** |  |  |
| --- | --- | --- |
| 1.   Replacement cost of the device/ laboratory ware |  |  |
| 2.   Its usefulness lifetime (hours, or years if used 3-4 hours daily) |  |  |
| 3.   Useful lifetime of battery, if any |  |  |
| 4.   Replacement cost of  the battery, if any ($ USD) |  |  |
| 5.   Approximate maintenance cost per year |  |  |
| 6.   Time needed for a test run |  |  |
| 7.   Skills needed for running the test and interpretation |  |  |
| 8.   Needed material and solvent and their corresponding costs per run |  |  |
| 9.   Any other requirement, such as, shipment and clearance cost, training, etc. |  |  |
| 10. Additional information |  |  |
| 11. Cost of light bulb replacement |  |  |
| 12. Consumable per sample |  |  |

Table A. Median total time taken per sample in sample set testing.

|  | **NIR-S-G1** | **MicroPHAZIR RX** | **TruScan RM** | **Progeny** | **4500a FTIR** | **PADs** |
| --- | --- | --- | --- | --- | --- | --- |
| **Median total time (seconds)** | 93.5 | 134 | 147.5 | 272.5 | 316 | 619.5 |

**Fig A. Results of sensitivity analyses from the cost-effectiveness analysis.**

One-way sensitivity analysis with different plausible parameter values in low prevalence scenario for (A) TruScan RM, (B) MicroPHAZIR RX, (C) 4500a FTIR, (D) Progeny, and (E) PADs (blue bars represents scenarios with lower parameter estimates, orange bars represent scenarios with the higher parameter estimates, and dotted vertical line represents the cost-effectiveness threshold)

A

B


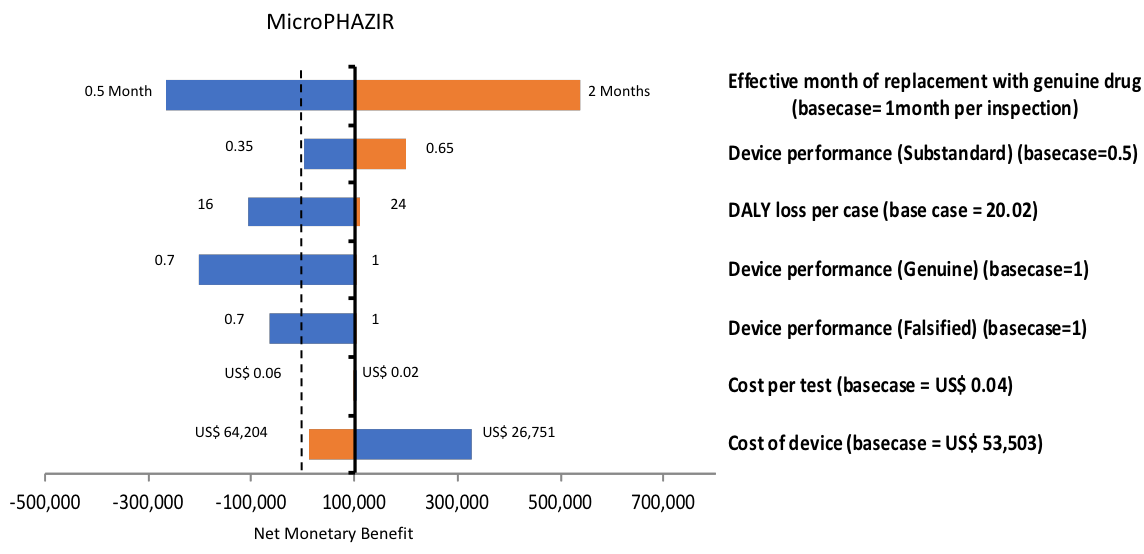


C


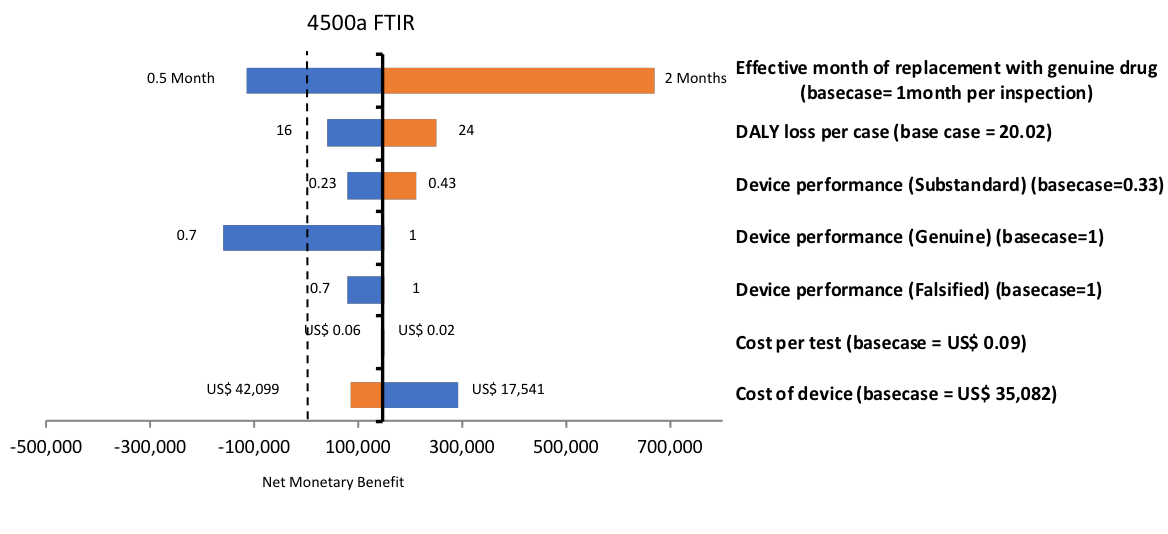


D

E

**Table B. Results from sensitivity analyses with lower detection rate with visual inspection (A) 12.5% and 25% reduction for substandard and falsified medicines, respectively and (B) 5% and 10% reduction under both high and lower prevalence scenarios.**

**(A) 12.5% and 25% reduction for substandard and falsified medicines with visual inspection;**

**i) High prevalence scenario**

| **Device** | **Cost** | **DALYs** | **Incremental Cost** | **DALY averted** | **ACER** | **INMB** |
| --- | --- | --- | --- | --- | --- | --- |
| **Baseline** | 81,900 | 1,445.2 |  |  |  |  |
| **NIR-S-G1** | 426,239 | 549.1 | 344,339 | 896.1 | 384 | 1,764,230 |
| **MicroPHAZIR RX** | 920,360 | 389.1 | 838,460 | 1,056.1 | 794 | 1,646,638 |
| **4500a FTIR** | 718,610 | 518.8 | 636,710 | 926.4 | 687 | 1,543,200 |
| **TruScan RM** | 1,026,706 | 454.0 | 944,806 | 991.3 | 953 | 1,387,698 |
| **PADs** | 279,016 | 978.3 | 197,116 | 466.9 | 422 | 901,559 |
| **Progeny** | 924,743 | 713.4 | 842,843 | 731.9 | 1,152 | 879,286 |

**ii) Lower prevalence scenario**

| **Device** | **Cost** | **DALYs** | **Incremental Cost** | **DALY averted** | **ACER** | **INMB** |
| --- | --- | --- | --- | --- | --- | --- |
| **Baseline** | 81,900 | 555.9 |  |  |  |  |
| **NIR-S-G1** | 202,880 | 267.0 | 120,980 | 289 | 419 | 558,755.65 |
| **4500a FTIR** | 486,888 | 259.4 | 404,988 | 296 | 1,366 | 292,584 |
| **MicroPHAZIR RX** | 664,783 | 194.6 | 582,883 | 361 | 1,613 | 267,282 |
| **PADs** | 150,206 | 439.1 | 68,306 | 117 | 585 | 206,363 |
| **TruScan RM** | 783,057 | 227.0 | 701,157 | 329 | 2,132 | 72,712 |
| **Progeny** | 728,801 | 356.7 | 646,901 | 199 | 3,248 | (178,220) |

**(B) 5% and 10% reduction for substandard and falsified medicines with visual inspection;**

**i) High prevalence scenario**

| **Device** | **Cost** | **DALYs** | **Incremental Cost** | **DALY averted** | **ACER** | **INMB** |
| --- | --- | --- | --- | --- | --- | --- |
| **Baseline** | 81,900 | 1,645.4 |  |  |  |  |
| **NIR-S-G1** | 481,257 | 599.7 | 399,357 | 1,045.7 | 382 | 2,061,167 |
| **MicroPHAZIR RX** | 981,698 | 422.5 | 899,798 | 1,222.9 | 736 | 1,977,684 |
| **4500a FTIR** | 775,860 | 563.3 | 693,960 | 1,082.1 | 641 | 1,852,176 |
| **TruScan RM** | 1,086,000 | 492.9 | 1,004,100 | 1,152.5 | 871 | 1,707,709 |
| **Progeny** | 975,859 | 774.5 | 893,959 | 870.9 | 1,027 | 1,155,157 |
| **PADs** | 313,366 | 1,085.0 | 231,466 | 560.3 | 413 | 1,086,944 |

**ii) Lower prevalence scenario**

| **Device** | **Cost** | **DALYs** | **Incremental Cost** | **DALY averted** | **ACER** | **INMB** |
| --- | --- | --- | --- | --- | --- | --- |
| **Baseline** | 81,900 | 622.6 |  |  |  |  |
| **NIR-S-G1** | 218,679 | 290.7 | 136,779 | 332 | 412 | 644,025 |
| **4500a FTIR** | 503,245 | 281.6 | 421,345 | 341 | 1,236 | 380,863 |
| **MicroPHAZIR RX** | 683,185 | 211.2 | 601,285 | 411 | 1,462 | 366,596 |
| **PADs** | 158,793 | 482.5 | 76,893 | 140 | 549 | 252,709 |
| **TruScan RM** | 800,436 | 246.4 | 718,536 | 376 | 1,910 | 166,508 |
| **Progeny** | 742,091 | 387.3 | 660,191 | 235 | 2,806 | (106,494) |

**Table C. Results from a** **sensitivity analysis when using one device per province instead of one per district per device in high prevalence scenario with a 1-sample strategy compared with visual inspection (ACER - average cost-effectiveness ratio) in descending order of net monetary benefit (NMB, US$).**

| **Name** | **Cost** | **DALYs** | **Incremental Cost** | **DALY averted** | **ACER** | **NMB** |
| --- | --- | --- | --- | --- | --- | --- |
| Baseline | 81,900 | 1111.73 |  |  |  |  |
| Microphazir RX | 97,396.32 | 333.52 | 15,496.32 | 778.21 | 19.91 | 1,815,629 |
| TruScan RM | 110,462.33 | 389.10 | 28,562.33 | 722.62 | 39.53 | 1,671,768 |
| 4500a FTIR | 74,189.90 | 444.69 | (7,710.10) | 667.04 | n/a | 1,577,246 |
| NIR-S-G1 | 39,826.29 | 464.90 | (42,073.71) | 646.82 | n/a | 1,564,048 |
| Progeny | 99,946.55 | 611.45 | 18,046.55 | 500.28 | 36.07 | 1,159,105 |
| PAD | 26,400.86 | 800.44 | (55,499.14) | 311.28 | n/a | 787,949 |
