# Supplementary material for: Implementation of field detection devices for antimalarial quality screening in Lao PDR—A cost-effectiveness analysis
Source: PLoS Negl Trop Dis. 2021 Sep 30;15(9):e0009539. doi: 10.1371/journal.pntd.0009539 (PMC8483304; doi:10.1371/journal.pntd.0009539)
Supplement: Attachment — Submitted filename: Reviewer report .docx [file pntd.0009539.s002.docx]

The authors sought to determine the Cost-Effectiveness of Field Detection Devices in Medicine Quality Screening in Lao PDR. Indeed, Substandard and falsified medicines pose a significant threat to the health system nowadays.

The authors did an outstanding work which add knowledge to the scientific community.

The methods used, the statistical analysis and data presentation is well indicated. The English write-up was also good.

It is a breakthrough to assess the cost-effectiveness of intervention methods to mitigate the impact of SF ACTs.

Listed below are minor comments:

- Your study focused on market surveillance among pharmacies in Laos. The study could have been more plausible if it is multicentered including various facilities to draw better concluding remarks.
- You have tested about 6 different devices to evaluate cost-effectiveness; however, device specific conclusions were not made lastly.
- The discussion is poorly written; It looks simply a repetition of your methodology and result part. Moreover, it states facts and I didn’t see comparisons with other similar studies. Suggestion: better to mention a portion of your result, compare with other similar (may not be exactly same) reports, then draw justification.
