# Supplementary material for: Implementation of field detection devices for antimalarial quality screening in Lao PDR—A cost-effectiveness analysis
Source: PLoS Negl Trop Dis. 2021 Sep 30;15(9):e0009539. doi: 10.1371/journal.pntd.0009539 (PMC8483304; doi:10.1371/journal.pntd.0009539)
Supplement: Attachment — Submitted filename: Response to reviewers_MedQualScreening.docx [file pntd.0009539.s003.docx]

**Responses to comments for the manuscript entitled ‘Implementation of Field Detection Devices in Medicine Quality Screening in Lao PDR – A Cost-Effectiveness Analysis’.**

**Manuscript ID:**  PNTD-D-20-02212

**Response to “Key Review Criteria Required for Acceptance?”**

**Reviewer #1 Comment#1:**

First, I think that te paper needs to more clearly present the objectives of the study rather than only saying that they are evaluating the cost-effectiveness of introducing the screening devices.  They should state what the testable hypothesis is for the study, the perspective of the study, and the target audience.  Also, since the study focused on testing of ACTs, it appears to be focused on antimalarials rather than other types of drugs.  In that case, the title of the study should be changed to reflect the focus on antimalarials as opposed to all medicines.  Iis not clear whether the findings can be generalized to other types of medicines.

**Response**

Thank you for your reflections. We have revised the text in the introduction to clearly present the objective, perspective and target audience in line 123 page 5 as “This study aims to evaluate the costs and consequences of implementing these six portable devices for the screening of antimalarials during pharmacy inspections and assess whether investment on these devices would be cost-effective from a healthcare provider’s perspective in the Laos setting.”. We didn’t see a need to present a testable hypothesis given the methodology used.

We agree that the title should relate more specifically to antimalarials and revised it to “Implementation of Field Detection Devices for Antimalarial Quality Screening in Lao PDR- A Cost-Effectiveness Analysis”.

**Reviewer #1 Comment#2:**

It would also be helpful to describe the prevalence and incidence of malaria in Lao PDR what interventions are currently being implemented against malaria, etc.

**Response**

We have added the information about incidence and prevalence of malaria as well as current intervention against malaria in Lao PDR in line 152 page 6: “The incidences of malaria cases and deaths in Lao PDR were reported as 10.2 cases per 1,000 population and less than 50 deaths in 2015 [20]. WHO reported the number of confirmed malaria cases in Laos in 2015 as 36,056 (Table 2). Almost all falciparum malaria cases in Laos are concentrated in 42 districts across five southern provinces (Savannakhet, Salavan, Sekong, Champasak, and Attapeu). Patients are assumed to be equally distributed across the districts and have equal access to 10 pharmacies per district. The key intervention to reduce the burden of malaria is the provision of long-lasting insecticide nets [21].”

**Reviewer #1 Comment#3:**

The study design is described but focuses largely on the assumptions of the decision analysis and effectiveness of the screening devices.  It requires more detail on data collection and costing methods.  It is unclear whether actual data collection was conducted in Lao PDR and, if so, who conducted the analysis.  Also, it does not include a description of the cost categories and costing methods used.  It does not appear that capital costs were depreciated as would be expected.  Also, the total costs of introducing the devices is not described, i.e. implementation costs as well as the costs of the devices, are not presented except in Table 7.  It would be good to see a table with total costs as well as proportions spent on each cost category since delivery costs are important in making decisions on introduction of a new technology.

**Response**

We have now included the details on data collection, costing methods and description of the cost categories and methods used in page 9 from line 207: “The total cost of implementing the screening devices was composed of costs for procurement and delivery, inspector visits, sample test reagents, reference testing by HPLC, and the cost of drug replacement if a tested sample failed the screening test. A micro-costing approach was used to estimate these costs. To estimate the cost of devices and delivery, fixed and variable costs for the devices and their consumables were collected from either manufacturer responses to a list of questions sent by email or the supplier quotation (S1 Text).”

In addition, we also present in the revised manuscript the separated costs between device and implementation costs as well as the proportion spent on each cost category in Table 4 (Page 12) and Figure 2 (Page 22).

**Reviewer #1 Comment#4:**

I found Table 1 difficult to interpret.  It would help to explain this table in more detail - e.g. what device fail and device pass means.  This information is in the decision analytic model but should also be stated in the table.

**Response**

We have further explained Table 1 in line 186-187 page 7 as “**^†^**Device:Fail refers to the probability that the device indicates that the medicine is substandard or falsified. **^‡^**Device:Pass refers to the probability that the device indicates that the medicine is genuine.”

**Reviewer #2:**

Yes, the methods used, the statistical analysis and data presentation is well indicated. The study met its objectives with sufficient sample size.

**Response**

Thank you for your reflections

**Reviewer #3 Comment #1:**

Use Strategies instead of overview heading.

In the parameter inputs section of the methods, it is better to include subtitles such as probabilities, costs and consequences, which in each section, how to calculate each item is explained in detail.

**Response**

Thank you for your reflections. We have now changed the heading to “Strategies” on page 5 line 133. We also included the subtitles; “Probabilities”, “Costs” and “Consequences” in the methods section and we further explain how each item was calculated in page 9 from line 207 of the revised manuscript as “The total cost of implementing the screening devices was composed of costs for procurement and delivery, inspector visits, sample test reagents, reference testing by HPLC, and the cost of drug replacement if a tested sample failed the screening test. A micro-costing approach was used to estimate these costs. To estimate the cost of devices and delivery, fixed and variable costs for the devices and their consumables were collected from either manufacturer responses to a list of questions sent by email or the supplier quotation (S1 Text).”

**Reviewer #3 Comment #2:**

Given that the measure of outcome is DALY averted in this study and the main parameters of DALY such as YLL and disability weight are assumed and there is no specific source or reference for them. Therefore, the results cannot be trusted.

**Response**

We have now described more details about assumption of disability weight and YLL for DALY estimates on page 9 line 232 as “These adverse outcomes are converted into disability adjusted life years (DALYs), using the assumption that the event of illness due to severe malaria would last for six weeks with 0.133 disability weight [23] and the average age of patients infected with malaria was conservatively assumed to be 48 years. Therefore, those who died of malaria would lose 20 life-years, based on the average life expectancy in Laos [24]. (Table 2)”

**Reviewer #3 Comment #3:**

The perspective of cost calculating, time horizon and discount rate are not mentioned in the method.

**Response**

The perspective, time horizon and discounting rate of this analysis were added on page 5 line 140 as “This analysis estimates the incremental costs of the six devices if incorporated within these inspection visits, and their outcomes measured in disability adjusted life years (DALYs) averted, taking a healthcare provider’s perspectives with a one year timeframe without discounting.”

**Response to “Results”**

**Reviewer #1:** The analysis presented matches the analysis plan to some extent. However, as noted above, it does not provide sufficient information on the costs of implementation and only presents this information in the budget impact.  This information should be presented in the general results section for the two scenarios and some charts developed with the shares of total costs for each cost category with and without the costs of the devices

**Response**

Thank you for this suggestion, we have now added the cost of implementation with those two scenarios in the general results section. The tables and pie charts with the percentage shared of total costs (with and without the costs of devices) were provided in Table 4 (page 12) and Fig. 2 (page 22) as well as in the main text in line 283 page 11 as “To implement the post-market surveillance with these devices nationally with a 1-sample strategy would incur an initial cost ranging between US$5,308 to US$2,893,292 in both prevalence scenarios (Table 4). The TruScan RM had the highest total initial cost followed by Progeny, MicroPHAZIR RX, 4500a FTIR, NIR-S-G1 and PADs, respectively. The total annual costs ranged from US$220,706 to US$380,317 under the high prevalence scenario and from US$139,925 to US$196,302 under the lower prevalence scenario. The MicroPHAZIR RX had the highest annual costs followed by the TruScan RM, 4500a FTIR, NIR-S-G1, PADs, and Progeny.

The procurement cost of devices is the largest cost category for all devices under both scenarios (Figure 2a and 2c) except NIR-S-G1 and PADs, where the largest cost category is the cost of reference testing and the cost of inspections, respectively under the high prevalence scenario (Figure 2a) and the cost of inspectors for both devices under the lower prevalence scenario (Figure 2c). Without accounting for the cost of devices, the reference testing cost category is the largest for all devices under high prevalence scenario except for PADs where the cost of inspections is the largest category (Figure 2b). The cost of inspections are the largest cost category for all devices under the lower prevalence scenario except NIR-S-G1 where the cost of reference testing is highest (Figure 2d).”

**Reviewer #2:** Yes

**Response**

Thank you.

**Reviewer #3:** The ACER column in Tables 4 and 5 should be changed to ICER because ACER is calculated for a strategy while the numbers in this column are incremental cost/ incremental effect for a strategy compare with baseline.

Probability sensitivity analysis(PSA) should be performed according to its importance in policy decisions

**Response**

Thank you for your reflection. We have now changed the column headings in the Tables to ICER.

We appreciate that PSAs are increasingly standard practice in cost-effectiveness analyses for health interventions, however we see limited added value for it in this instance, where we are providing only a general and conservative assessment of the potential cost-effectiveness of these devices. This is sufficient to confirm that from a health economics viewpoint, the implementation of these devices, including the more costly ones, could represent good (health) returns on investment, even when focusing only on their use for antimalarials. As we state in the conclusion – once stakeholders are on board with the potential use of such devices for post market surveillance, more contextualized and detailed analyses will be required to identify the optimal devices and strategies for specific settings, for which PSAs will be essential.

**Response to “Conclusion”**

**Reviewer #1:** In the discussion section, it would be helpful to compare the findings with other studies in the literature if there are any.  Also, it would be good to compare the delivery costs with and without the cost of devices with current expenditures on ACT screening if this information is available.

**Response**

There are few similar studies in the literature, all focused on estimating the burden of poor quality medicine and one assessed the economic impact of possible intervention. However, none of them evaluated the cost-effectiveness of devices to detect medicine quality in field inspections. Therefore, we could only provide additional information about these studies and include the generalisability of our findings to other countries in the discussion section line 453 page 18 as, “The number of previous studies estimating the burden and economic impact of poor quality antimalarials in low- and middle-income countries is very limited [28-30] and only one of these assessed the economic impact of interventions that might address this impact, focusing on eliminating antimalarial stock-outs, increasing care seeking rates for malaria treatment, and encouraging better adherence to antimalarial treatment [26]. The annual loss due to SF antimalarials was estimated to be US$ 892 million in Nigeria [28] and at US$ 614 million for the population aged under five in Uganda [30]. Our work is one of the first attempts to evaluate the cost-effectiveness of interventions to mitigate the impact of SF ACTs (and SF medicines more broadly) and to our knowledge the first to consider the cost-effectiveness of devices to detect medicine quality in field inspections. We based our analysis in the context of Laos, but our findings might be informative for other countries where malaria endemicity and the prevalence of poor quality medicine in considering these devices for post-market surveillance.”

Moreover, we also added the information when comparing the delivery costs and cost of device with the current expenditures on visual screening in line 404, page 17 as “Our findings show that for the majority of devices, the initial procurement cost alone is much higher than the annual cost of visual ACT inspection alone (ranging from 18 times higher with the 4500 Agilent to 35 times higher with TruScan RM), with the exception of PADs and NIR-S-G1where the costs of device are lower. This could act as a deterrent from their adoption, despite being potentially highly cost-effective.”

**Reviewer #2:** Discussion is poorly written.

**Response**

We have improved the writing of the discussion.

**Reviewer #3:** (No Response)

**Response**

N/A

**Response to “Editorial and Data Presentation Modifications?”**

**Reviewer #1:** (No Response)

**Response**

N/A

**Reviewer #2:** Minor revision

**Response**

N/A

**Reviewer #3:** (No Response)

**Response**

N/A

**Response to “Summary and General Comments”**

**Reviewer #1:** The paper discusses the cost-effectiveness of of portable screening devices to assess the quality of medicines in Lao PDR.  The paper indicates that insufficient screening of medicine quality is being conducted, particularly to assess substandard medicines.  However, the paper needs to improve its situation analysis, presentation of methods and results, as well as discuss other literature on the subject.  It is unclear whether the study is focusing on antimalarials rather than all medicines, the title does not seem to match the content, and the costing methods are not well-described and presented.

**Response**

We have now improved the writing in all sections including the presentation of costing methods, results and other comparison with other studies. We also revised the title of this study to better reflect the focus on antimalarial medicines, as mentioned earlier in this document.

**Reviewer #2 Comment #1:** The authors sought to determine the Cost-Effectiveness of Field Detection Devices in Medicine Quality Screening in Lao PDR. Indeed, Substandard and falsified medicines pose a significant threat to the health system nowadays.

The authors did an outstanding work which add knowledge to the scientific community.

The methods used, the statistical analysis and data presentation is well indicated. The English write-up was also good.

It is a breakthrough to assess the cost-effectiveness of intervention methods to mitigate the impact of SF ACTs.

Listed below are minor comments:

•    Your study focused on market surveillance among pharmacies in Laos. The study could have been more plausible if it is multi-centered including various facilities to draw better concluding remarks.

**Response**

Thank you for this reflection. We agree it would be very informative to consider the impact of using these devices in different contexts. Our focus on PMS in private sector pharmacies is explained by this sector being generally less well regulated, and where PMS is most critical.

**Reviewer #2 Comment #2:**

•    You have tested about 6 different devices to evaluate cost-effectiveness; however, device specific conclusions were not made lastly.

**Response**

We provide conclusion about the devices and the screening strategy based on the findings from this cost-effectiveness study in the first paragraph of the discussion section. Other aspects to take into account to choose the right device (e.g. accuracy and usability) are provided in the other manuscripts within the same PLOS collection of articles on our collaborative multi-phases study.

**Reviewer #2 Comment #3:**

•    The discussion is poorly written; It looks simply a repetition of your methodology and result part. Moreover, it states facts and I didn’t see comparisons with other similar studies. Suggestion: better to mention a portion of your result, compare with other similar (may not be exactly same) reports, then draw justification.

**Response**

We hope the changes mentioned above to the discussion improve its reading. As explained above, there are no comparable studies looking at the cost-effectiveness of PMS devices.

**Reviewer #3 Comment #1:**

**:** In the abstract, instead of the analysis scenario, it is better to emphasize the type of costs that have been calculated and the outcome measurement index.

**Response**

We have now added the type of costs and outcome measurement index in the abstract as “Costs of inspection including device procurement, inspector time, reagents, reference testing, and replacement of failing samples with genuine ACTs were estimated. Outcomes were measured as disability adjusted life years (DALYs).”

**Reviewer #3 Comment #2:**

Depending on the type of economic evaluation study (cost utility analysis), it is better to use the term outcome or effect instead of benefit in all parts of the manuscript.

**Response**

We have now used term “outcome” in all parts of the manuscript.
