# Supplementary material for: Implementation of field detection devices for antimalarial quality screening in Lao PDR—A cost-effectiveness analysis
Source: PLoS Negl Trop Dis. 2021 Sep 30;15(9):e0009539. doi: 10.1371/journal.pntd.0009539 (PMC8483304; doi:10.1371/journal.pntd.0009539)
Supplement: Attachment — Submitted filename: Response to reviewers_R2 PLoS Neg_.docx [file pntd.0009539.s004.docx]

**Responses to comments for the manuscript entitled ‘Implementation of Field Detection Devices in Medicine Quality Screening in Lao PDR – A Cost-Effectiveness Analysis’.**

**Manuscript ID:**  PNTD-D-20-02212R1

**“Methods”**

**Reviewer #1:**

The objectives of the study are clearly stated. The study design seems appropriate.  In general, the methods seem appropriate.  I question, though, why the study authors did not use discounting for their capital costs.  In a cost-effectiveness study, it is recommended to estimate economic costs rather than financial costs and the capital costs should be discounted.  So, I suggest that the authors should discount the capital costs.

**Response**

Thank you for your suggestion. In this study, we used the one year timeframe to evaluate both the cost and the impact of all medicine screening devices. We conservatively assumed that these devices have a useful life of five years therefore applied an annual cost equivalent to 1/5^th^ of their actual procurement costs. With the one year timeframe and short useful life assumption, we think this annualization of the costs is sufficient, with little added value by introducing discounting, maintenance, etc..

**Reviewer #3:** (No Response)

**“Results”**

**Reviewer #1:** (No Response)

**Reviewer #3:** (No Response)

**“Conclusions”**

**Reviewer #1 Comment#1:**

I have some comments about the conclusions. First, for the budget impact analysis, I don't think it makes sense to say that donor support to purchase some of the devices would be required since that would not be supporting sustainability.  Instead, it would be better to say what the most affordable options are, given the available budget.

**Response**

Thank you for your reflection. We agree with the reviewer that the donor support would not be a sustainable approach, therefore we have removed this sentence and added the suggested information on page17 line 403 as “The budget impact analysis also suggests that some of these devices could be affordable for local authorities, in particular the 4500 Agilent, NIR-5-G1, and PADs.”

**Reviewer #1 Comment#2:**
It seems obvious that cost-effectiveness is not an 'inherent' feature of the devices since cost-effectiveness will vary with the probability of SF, the costs, and effectiveness.  So I would reword that sentence.

**Response**

We have now revised the sentence on page 17 line 411 as “While in many of the scenarios the devices appeared to be cost-effective, it is important to emphasize that cost-effectiveness is not is to a great extent contextual and will depend on how the devices are implemented and how their use alters pre-existing practices.” as suggested.

**Reviewer #1 Comment#3:**
I also think that it would be useful to discuss whether there are any other uses of the devices that would increase their cost-effectiveness.

**Response**

We have now added the other uses of the devices to increase their cost-effectiveness on page 18 line 442 as “, particularly antimicrobial agents such as amoxicillin, acyclovir, doxycycline, ethambutol and isoniazid that can cure many widely prevalent infections, and ensuing genuine medicines are used can help slow the development of antimicrobial resistant to these agents in the long run [28, 29]”.

**Reviewer #3:** (No Response)

**“Summary and General Comments”**

**Reviewer #1:** In general, the revised paper has improved.

**Response**

Thank you for your reflections.

**Reviewer #3:** (No Response)
