# Supplementary material for: Implementation of field detection devices for antimalarial quality screening in Lao PDR—A cost-effectiveness analysis
Source: PLoS Negl Trop Dis. 2021 Sep 30;15(9):e0009539. doi: 10.1371/journal.pntd.0009539 (PMC8483304; doi:10.1371/journal.pntd.0009539)
Supplement: Attachment — Submitted filename: Response to reviewers_R3 PLoS Neg_final.docx [file pntd.0009539.s006.docx]

**Responses to comments for the manuscript entitled ‘Implementation of Field Detection Devices in Medicine Quality Screening in Lao PDR – A Cost-Effectiveness Analysis’.**

**Manuscript ID:**  PNTD-D-20-02212R3

**Response to Comments**

**Comment #1:** I see that the authors added a paragraph on malaria incidence to the paper.  However, I think that it should be in the introduction section rather than the methods section.  Also, they should say 'incidence' rather than 'incidences.'  I also think that they should add a sentence on the burden of malaria illness compared to other diseases and why it would be important to ensure that ACTs are of high quality and not SF.

**Response**

Thank you for your suggestion. We’ve moved the paragraph on malaria incidence to the introduction section and revised the term to ‘incidence’. We also added a sentence comparing the burden of malaria to other diseases and this is all now placed just before a sentence stating why the high quality of ACTs is important: “Despite declines in transmission, malaria is still ranked fourth in amongst infectious diseases in terms of its global burden, following diarrheal diseases, HIV, and tuberculosis, respectively [9]. The incidence of malaria cases and deaths in Laos were reported as 10.2 cases per 1,000 population and less than 50 deaths in 2015 [10]. WHO reported the number of confirmed malaria cases in Laos in 2015 as 36,056 [10]. Malaria is curable but in the absence of effective treatment can be fatal. Artemisinin-based combination therapies (ACT) are now recommended as first-line therapy for *Plasmodium falciparum* malaria worldwide [11]. A recent systematic review suggested that SF antimalarial drugs, including ACTs, are widely distributed across LMICs with a mean prevalence of 19.1% [12]. The distribution of SF ACTs endangers patients and in the longer term can threaten malaria control and elimination campaigns by promoting the development of drug resistance [13-15].”

**Comment #2:** The phrasing of some sentences is awkward.  In several places, it would be preferable to use the term 'introducing' rather than 'implementing.  - e.g. pg. 5, line 123.

**Response**

We’ve used the term ‘introducing’ where relevant, including page 2 line 43, page 5 line 127, page 9 line 209, page 10 line 267, page 18 line 433, and page 22 line 531-536.
